# Synthesis and Verification of Biobased Terephthalic Acid from Furfural

**DOI:** 10.1038/srep08249

**Published:** 2015-02-04

**Authors:** Yuya Tachibana, Saori Kimura, Ken-ichi Kasuya

**Affiliations:** 1 Division of Molecular Science, Faculty of Science and Technology, Gunma University, 1-5-1 Tenjin, Kiryu, Gunma 376-8515, Japan; 2 PRESTO, Japan Science and Technology Agency (JST), 4-1-8 Honcho, Kawaguchi, Saitama 333-0012, Japan

## Abstract

Exploiting biomass as an alternative to petrochemicals for the production of commodity plastics is vitally important if we are to become a more sustainable society. Here, we report a synthetic route for the production of terephthalic acid (TPA), the monomer of the widely used thermoplastic polymer poly(ethylene terephthalate) (PET), from the biomass-derived starting material furfural. Biobased furfural was oxidised and dehydrated to give maleic anhydride, which was further reacted with biobased furan to give its Diels-Alder (DA) adduct. The dehydration of the DA adduct gave phthalic anhydride, which was converted via phthalic acid and dipotassium phthalate to TPA. The biobased carbon content of the TPA was measured by accelerator mass spectroscopy and the TPA was found to be made of 100% biobased carbon.

As social demand for renewable sources of commodity plastics has increased, biomass has attracted much attention as a promising alternative to petrochemicals^1^,^2^,^3^. The resources used to produce biobased plastics should be inedible, waste, and abundant. Furfural is an ideal biomass resource, as it is traditionally produced from cellulosic and waste biomass such as corncob, corn stock, and rice hull. Furthermore, it is extremely abundant, with a global output of 500,000–1,000,000 tonne/year^4^,^5^. The US Department of Energy (DOE) has stated that it is one of the most value-added chemicals derived from biomass^6^.

Another advantage of using furfural is that it can be readily converted to other useful chemicals due to its high reactivity^7^. Furfural is industrially converted to furan via decarboxylation and has been used as a source of bio-fuels^7^,^8^,^9^,^10^. In previous works, we have synthesised succinic acid and 1,4-butanediol from furfural, and polymerised them to the biodegradable thermoplastic resin poly(butylene succinate)^11^,^12^,^13^. We have further used it to produce biobased acid anhydrides and polymerised them with diols to polyoxabicyclates, which are a potential alternative to commercially available transparent elastic polymers^14^.

In this work, we have focused on producing biobased poly(ethylene terephthalate) (PET). PET resin is a widely used commodity plastic with applications in fibre manufacture, packaging, and electric devices. Coca-Cola Ltd. and other beverage companies have adopted biobased-PET since 2009 when they announced that their containers would change from petroleum-based PET to biobased PET^1^,^15^,^16^. However, this commercially available biobased PET is composed of bio-ethylene glycol derived from bio-ethanol, and petroleum-based terephthalic acid (TPA) made from *p*-xylene produced by fractional distillation of naphtha. Consequently, its percentage biomass carbon content, as defined below, is a mere 20%.

The development of a route to biobased TPA would allow the synthesis of fully biobased PET, an extremely attractive prospect from both economic and environmental viewpoints. Consequently, many researchers have attempted to develop a viable method to produce biobased TPA and its precursors. *p*-Xylene, a precursor of TPA, has been obtained from two different biomass resources: glyceride, supplied as a by-product of commercially available chemicals such as bio-diesel; and 5-hydroxymethyl-2-furfural, derived from cellulosic waste^17^. The monoterpene *p*-cymene has been directly oxidised to TPA^18^, and ethylene, which can be produced from bio-ethanol, has been converted to *p*-xylene^19^. The conversion of biomass chemicals such as ethylene, isobutene, furan, and dimethylfurfural to *p*-xylene using heterogeneous catalysts has also been investigated^20^,^21^,^22^,^23^,^24^. Furthermore, Toray industries Inc. launched the production of a fully biobased PET fibre with biobased TPA derived from isobutene in 2012^25^,^26^.

In this work, we propose a viable synthetic route, shown in Figure 1, for the preparation of biobased TPA from furfural alone, which is produced industrially from inedible cellulosic biomass. In addition, we verified that the TPA obtained was indeed biobased, as determined by the measurement of the biobased carbon content. Biobased carbon content has been adopted as a criterion for biomass-sourced chemicals in several international standards such as ISO, ASTM, and EN as it is the most practical and effective method for verifying that a material contains carbon derived from biomass^27^,^28^,^29^. It is determined by measuring the value of the ^14^C/^12^C ratio in the material, and is based on the assumption that this ratio for petroleum-derived (or ‘ancient’) carbon is 0, while the ratio for biobased (or ‘modern’) carbon is 1 × 10^−12^. In our work, this ratio was measured using accelerator mass spectroscopy (AMS) according to guidelines listed in ISO 16620-2. The details of the measurement procedure have been previously reported in the literature^28^.

## Results and Discussion

### Oxidation of furfural to fumaric acid and maleic acid

The oxidisation of furfural with NaClO_4_ as an oxidant, and V_2_O_5_ as a catalyst, gave a mixture of fumaric acid and maleic acid in 58% yield, lower than the 72% yield reported in the literature^30^. This lower yield could be caused by vigorous oxidation, which is difficult to control. The oxidation of furfural to maleic acid and fumaric acid has been performed by other means with improved yields and better selectivity for the products, either maleic acid or fumaric acid, elsewhere in the literature^31^,^32^. However, the oxidation with NaClO_4_ is, for our purposes, a more practical laboratory process, and, therefore, we adopted this traditional oxidation method in this study.

### Dehydration of fumaric acid and maleic acid to maleic anhydride

The dehydration of the mixture of maleic acid and fumaric acid to maleic anhydride was performed using P_2_O_5_ as a dehydration agent. As the ratio of maleic acid and fumaric acid, determined by ^1^H NMR, was 1:7, the yield of maleic anhydride could potentially be less than 30%. However, once maleic acid was dehydrated to give maleic anhydride, phosphoric acid, produced by the reaction of P_2_O_5_ and water, could isomerise fumaric acid to maleic acid^33^. Consequently, the mixture of maleic acid and fumaric acid was quantitatively converted to maleic anhydride.

### Diels-Alder (DA) reaction of anhydrous maleic acid and furan to the *exo*-DA adduct

The synthesis of furan from furfural could have been demonstrated in this study. However, commercially available furan is a biobased chemical and has been verified as such in previous work^13^. The biobased carbon contents of the furan and furfural used in this study are shown below. Therefore, although we have not performed the process ourselves, for the purposes of this work, commercially available furan is assumed to be biobased.

The DA reaction of maleic anhydride and furan readily gave the DA adduct. At the beginning of the reaction, the regioselectivity of DA cyclisation is for the *endo*-adduct due to its kinetic stability. However, the DA reaction is reversible and, after some time, the product is converted to the more thermally stable *exo*-DA adduct^34^,^35^,^36^. Consequently, the reaction was carried out for 12 h at room temperature, yielding the DA adduct in almost quantitative yield. Melting point analysis showed the m.p. of the product to be 127–129°C, corresponding to the *exo* adduct.

### Dehydration of the *exo*-DA adduct to phthalic anhydride

The oxabicyclo moiety in the *exo*-DA adduct is readily dehydrated with an acid. We attempted to dehydrate the *exo*-DA adduct using sulfonic acid, phosphoric acid, and P_2_O_5_, but the yield and purity of the phthalic anhydride produced were not high enough to isolate it. However, a more effective protocol for the dehydration of the *exo*-DA adduct has been recently developed in which it is treated with a mixture of trifluoromethane sulfonic acid and acetic anhydride^37^. We employed this new method and obtained phthalic anhydride in 84% yield.

### Hydrolysis of phthalic anhydride to dipotassium phthalate

Phthalic acid was readily hydrolysed with aqueous potassium hydroxide to give dipotassium phthalate quantitatively.

### Transfer reaction and acidification of dipotassium phthalate to TPA

Half a century ago, a transfer reaction known as the Henkel method, which converts dipotassium phthalate to dipotassium terephthalate at high temperature (above 400°C) with CdI_2_ as a catalyst, was the most common industrial process for the production of TPA^38^,^39^. The development of an alternative method involving the oxidation of *p*-xylene to TPA led to the Henkel method losing its competitive advantage and falling out of favour. Consequently, it is rarely used industrially. However, in this study, we adopted the Henkel method to convert biobased phthalate to biobased TPA, as we found it to be a practical method for obtaining biobased TPA from furfural. The reaction was carried out with CdI_2_ at 420°C, and the resulting mixture was acidified to give biobased TPA. At 44%, the yield of TPA obtained in this study is not sufficient. However, this figure is obtained at the milligram scale, but the process was optimised industrially, so, therefore, it is reasonable to expect that the yield would increase for industrial production. Additionally, the Henkel method is proven as an industrial process, and, therefore, the commercial viability of this synthetic route from furfural to TPA is already established.

### Biobased carbon content

The biobased carbon contents of the reagents and products are summarised in Table 1. The synthesis of fully biobased TPA is verified by the fact that the values of furfural, furan, and TPA were almost 100%. Thus, we can conclude that both the starting materials and product are fully biobased chemicals.

On the other hand, the values for furfural and furan reported previously were 100.8 and 105.0%^11^,^13^ and slightly higher than those measured in this study. These values were obtained in 2010, while those in this study were obtained in 2014. Since the lot numbers of furfural and furan used in this study are different from those used in the previous study, the actual value of the ^14^C/^12^C ratio could be slightly different. This difference could be explained by the difference in the definition of biobased carbon content between ISO 16620-2 and ASTM D6866 and the manufacturing year of furfural and furan. In principle, the percentage of modern carbon (pMC) calculated from the ^14^C/^12^C concentration ratios, is the biobased carbon content. However, the pMC for biomass produced by fixation of CO_2_ in the atmosphere by photosynthesis was 108–110% in 2002^27^,^28^,^29^. The pMC is possibly slightly higher than 100% because of the continuing but diminishing effects of nuclear testing in the atmosphere in the 1950s, during which large amounts of ^14^C were emitted into the atmosphere. Because the ^14^C in all the samples is referenced to a “prebomb” standard, i.e., modern carbon-based oxalic acid radiocarbon [Standard reference material (SRM) 4990c, National Institute of Standards, USA], all pMC values must be multiplied by a cofactor to correct for the bomb carbon and to obtain the true biobased carbon content of the sample. In our previous study, the biobased carbon contents were calculated using a strong cofactor of 0.93, that being the old value based on ASTM D6866 (2008) because the furfural and furan used were purchased before 2009. Nevertheless, the reason why the value of furan was above 105% is that it was produced before 2008. In this study, as the reagents used were purchased in 2013, the biobased carbon contents were calculated using the new value for the weak cofactor of 0.95 based on ISO 16620-2. These indicate that the nuclear testing effect on the old reagents was strong and the biobased carbon content was above 100%, even though the strong cofactor 0.93 was used, while the effect on the new reagents was weaker, giving a biobased carbon content of almost 100%. Therefore, the biobased carbon contents of furan and furfural in this study were slightly different from the values measured in the previous study.

The precise method for the measurement of biobased carbon content is detailed in ISO 16620-2 and ASTM D6866 and is an industrially indispensable verification procedure^29^,^40^. It is important, not just to prevent mistakes by researchers, but also to detect whether supposedly biobased materials have undergone some contamination from, or carbon exchange with, petrochemical sources such as other reaction reagents or non-biobased solvents. For example, in the case of the Henkel method, the transfer reaction could involve the incorporation of a carbonyl carbon from carbon dioxide, produced as a by-product of a petrochemical process^38^,^41^. In addition, biobased carbon content measurement is also an invaluable method for identifying materials mistakenly or falsely supplied as biobased. Therefore, we propose that the measurement of biobased carbon content should be necessary when biobased chemicals are used, especially when the products can be synthesised from commercially available petroleum-derived starting materials or involve the use of non-biobased reagents or solvents.

### In summary

We successfully synthesised biobased TPA from furfural and furan using viable and proven organic synthetic procedures. Furthermore, the biobased carbon content of the TPA that we synthesised confirmed that it is a truly biobased product. Using furfural as a single resource is a novel and interesting concept, since furfural can be produced from inedible cellulosic biomass.

The aim of this study was to propose a viable synthetic route from furfural alone to TPA, and we have succeeded in this. It is our hope that more research, conducted by both ourselves and, perhaps, other groups, will optimise this process so that it may be industrialised.

We finally propose that the measurement of biobased carbon content is indispensable as a verification method in the research area of biobased synthesis.

## Methods

### Materials

Furan, sodium chlorate, vanadium pentoxide, phosphorus pentoxide, methane sulfonic acid, acetic anhydride, cadmium iodide, toluene, and diethyl ether were purchased from Wako Pure Chemical Industries (Osaka, Japan). Furfural, potassium hydroxide, and hydrochloric acid were purchased from Kanto Kagaku Co., Inc (Tokyo, Japan). Phosphorus pentoxide was purchased from Kishida Chemical Co., Ltd (Osaka, Japan). Furfural, trifluoromethane sulfonic acid, and acetic anhydride were used after distillation under reduced pressure. All other chemicals were of reagent grade and used without further purification.

### Instrumentation

^1^H NMR spectra were recorded on a 400 MHz NMR spectrometer (JNM-ECX400; JEOL, Tokyo, Japan) using deuterated chloroform or deuterated dimethyl sulfoxide as a solvent, and tetramethylsilane as an internal standard.

### Measurement of biobased carbon content^28^

Measurements of the ratios of the three carbon isotopes (^14^C, ^13^C, and ^12^C) using AMS were performed at the Institute of Accelerator Analysis Ltd (IAA) (Fukushima, Japan) using a 3-MV tandem accelerator (National Electrostatics Co., Middleton, WI, USA, Pelletron 9SDH-2). The pMC was calculated from the ^14^C/^12^C concentration ratios for the sample. The biobased carbon content was determined from the ratio of ^14^C/^12^C concentrations of the sample according to ISO 16620-2. Δ ^14^C is the isotope differential ratio of ^14^C between the sample and reference material. Reference materials were also analysed using AMS. The biobased carbon content was calculated as follows: 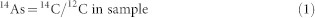

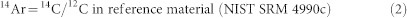

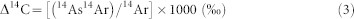

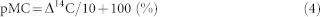

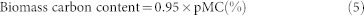


### Oxidation of furfural to fumaric acid and maleic acid^30^

Furfural (36 g, 367 mmol) was carefully added dropwise to a solution of sodium chlorate (80 g, 751 mmol) and vanadium pentoxide (360 mg, 1.98 mmol) in water (10 mL) at 90°C over 3 h, and the mixture was stirred at 80°C for a further 10 h. The mixture was allowed to stand at room temperature for 11 h, affording a white crystalline precipitate. The precipitate was filtered and dried to give 42.3 g (58%) of a mixture of fumaric acid and maleic acid as white crystals. ^1^H NMR (400 MHz, DMSO-*d*_6_) *δ* 6.61 (2H, s, –CH = of fumaric acid), 6.01 (2H, s, –CH = of maleic acid) ppm.

### Dehydration of fumaric acid and maleic acid to maleic anhydride^42^

The mixture of fumaric acid and maleic acid prepared above (3.00 g, 25.8 mmol) was mixed with diphosphorus pentoxide (5.00 g, 35.2 mmol) using a pestle and mortar. The resulting mixture was added to a sublimation apparatus and sublimated at 140°C and 0.4 kPa for 3 h. The resulting sublimate was collected to give 2.41 g (95%) of maleic anhydride as white crystals. ^1^H NMR (400 MHz, CDCl_3_) *δ* 7.46 (2H, s) ppm.

### Diels–Alder (DA) reaction of anhydrous maleic acid and furan to *exo-*DA adduct^34^

Maleic anhydride (4.65 g, 47.4 mmol) was dissolved in diethyl ether (50 mL). Furan (18 mL, 278 mmol) was added and the reaction mixture was allowed to stir at room temperature for 12 h. The resulting precipitate was collected by filtration to give 7.77 g (98%) of white crystals. The melting point of white crystals was 127–129°C. This indicates that the white crystals obtained is *exo-*DA adduct. ^1^H NMR (400 MHz, DMSO-*d*_6_) *δ* 6.58 (2H, s), 5.46 (2H, s), 3.18 (2H, s) ppm.

### Dehydration of *exo-*DA adduct to phthalic anhydride^37^

The *exo-*DA adduct (1.00 g, 6.02 mmol) was added to a mixture of methane sulfonic acid (10.0 mL, 154 mmol) and acetic anhydride (2.0 mL, 21 mmol) under a N_2_ atmosphere at 0°C. The reaction mixture was allowed to warm to room temperature and stirred for 2 h. It was then heated to 80°C and stirred for 4 h. After cooling to room temperature, the reaction mixture was extracted with toluene (3 × 20 mL). The combined toluene extract was washed with saturated sodium hydrogen carbonate solution and saturated sodium chloride solution before being dried over anhydrous sodium sulfonate. After filtration, the organic layer was evaporated *in* *vacuo* to give 746 mg (84%) of phthalic anhydride as white crystals. ^1^H NMR (400 MHz, CDCl_3_) *δ* 8.08–7.95 (2H, m), 7.65–7.52 (2H, m) ppm.

### Hydrolysis to phthalic anhydride to dipotassium phthalate^43^

Phthalic anhydride (500 mg, 3.38 mmol) was dispersed in a solution of potassium hydroxide (1.00 g, 17.8 mmol) in water (10 mL). The reaction mixture was allowed to stir at 80°C for 2 h. The resulting solution was poured into ethanol (150 mL) affording a white precipitate which was filtered to give 802 mg (98%) of dipotassium phthalate as white crystals. ^1^H NMR (400 MHz, D_2_O) *δ* 7.36–7.25 (4H, m, –CH = ) ppm.

### Conversion and acidification of dipotassium phthalate to TPA^43^

A mixture of dipotassium phthalate (800 mg, 3.30 mmol) and cadmium iodide (40 mg, 0.11 mmol) was ground in a pestle and mortar. The mixture was placed in a sealing tube and sealed *in vacuo*. After heating to 420°C for 2 h, the resulting solid was placed into hot water (30 mL). The dispersion was refluxed for 10 min and the remaining precipitate was removed by filtration. The filtrate was acidified with 1 M HCl and the resulting solid was filtered off to give 242 mg (44%) of a white solid. ^1^H NMR (400 MHz, CDCl_3_) *δ* 8.03 (4H, s, –CH = ) ppm.

## Author Contributions

Y.T. and K.K designed this work and wrote the paper. Y.T. and S.K. carried out the synthetic experiments. All the authors participated in analysis and discussion of the results.

## Figures and Tables

**Figure 1:**
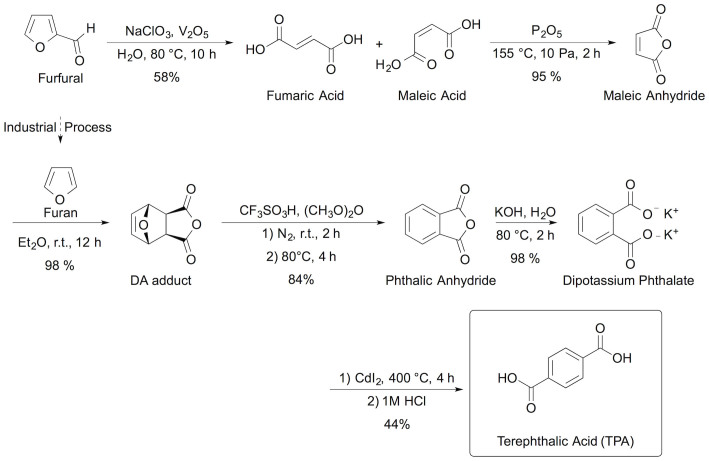
Synthetic route to biobased TPA from biomass-derived furfural. The furan used to produce the DA adduct is processed from the same biomass-derived furfural employed at the beginning of the route, making the product entirely biobased.

**Table 1:** Biobased carbon content of biobased chemicals calculated from Δ ^14^C measured by accelerated mass spectrometry (AMS), in accordance with ISO 16620-2

Chemicals	Δ ^14^C^a^ ‰	pMC^a^ %	biobased carbon content %
Furfural	44.59	104.46	99.2
Furan	51.96	105.20	99.9
Terephthalic Acid (TPA)	38.61	103.86	98.7

^a^Measured by AMS.
